# Neutrophil extracellular traps mediate the crosstalk between glioma progression and the tumor microenvironment ***via*** the HMGB1/RAGE/IL-8 axis

**DOI:** 10.20892/j.issn.2095-3941.2019.0353

**Published:** 2020-02-15

**Authors:** Caijun Zha, Xiangqi Meng, Lulu Li, Shan Mi, Da Qian, Ziwei Li, Pengfei Wu, Shaoshan Hu, Shihong Zhao, Jinquan Cai, Yanhong Liu

**Affiliations:** ^1^Department of Laboratory Diagnosis, The Second Affiliated Hospital of Harbin Medical University, Harbin 150086, China; ^2^Department of Neurosurgery, The Second Affiliated Hospital of Harbin Medical University, Harbin 150086, China; ^3^Neuroscience Institute, Heilongjiang Academy of Medical Sciences, Harbin 150086, China

**Keywords:** Neutrophil extracellular traps, HMGB1, IL-8, NF-κB, glioma microenvironment

## Abstract

**Objective:** Neutrophil extracellular traps (NETs) produced by tumor-infiltrating neutrophils (TINs) are associated with poor prognosis in patients with several types of cancer. However, the mechanisms underlying the involvement of NETs in glioma progression remain largely unknown. This study aimed to elucidate the roles of NETs in biological processes that drive the crosstalk between glioma progression and the tumor microenvironment.

**Methods:** Neutrophil infiltration and NETs formation were investigated in glioma tissue through immunohistochemistry, and their relationships with clinicopathological features and outcomes were statistically evaluated. The effects of NETs on glioma cell progression were studied in a co-culture system. *In vivo* and *in vitro* experiments validated the reactive oxygen species activity and cytokine production of TINs, as well as the ERK signaling pathway activation and the metastasis of gliomas.

**Results:** Neutrophil infiltration and NETs formation were induced in high-grade glioma compared with low-grade glioma. NETs induced by TINs were determined to be an oncogenic marker of high-grade gliomas and to be involved in cell proliferation and invasion. NETs overproduction promoted glioma cell proliferation, migration, and invasion. Furthermore, HMGB1 was found to bind to RAGE and activate the NF-κB signaling pathway *in vitro*. In addition, NETs stimulated the NF-κB signaling pathway, thus promoting IL-8 secretion in glioblastoma. Subsequently, IL-8 recruited neutrophils which in turn mediated NETs formation *via* the PI3K/AKT/ROS axis in TINs.

**Conclusions:** Our results suggest that NETs produced by TINs mediate the crosstalk between glioma progression and the tumor microenvironment by regulating the HMGB1/RAGE/IL-8 axis. Targeting NETs formation or IL-8 secretion may be an effective approach to inhibit glioma progression.

## Introduction

Gliomas, the most common malignant primary tumors of the brain and spinal cord in adults, are classified into 4 grades [World Health Organization (WHO) grade I to grade IV, from lowest to highest malignancy]^1^. Despite comprehensive treatment with surgical resection, chemotherapy, and radiotherapy, patients with glioblastoma (GBM; WHO grade IV) survive no more than 15 months on average and have a 5-year survival rate of only 5.5%^2^. The aberrant activation of inflammatory responses is partly accountable for the poor prognostic outcomes of GBM^3^. Unregulated chronic inflammation results in the production of growth factors and reactive oxygen species (ROS), which predispose individuals to tumor progression^4^.

The progression of tumors is closely related to the tumor microenvironment (TME), which is composed of various nontumor cells, secreted factors, signaling molecules, and extracellular matrix components^5^. The TME is emerging as a key regulator in the occurrence, progression, and invasion of brain tumors. Cytokines and chemoattractants secreted by tumor cells recruit immune cells to the TME, and those immune cells in turn supply pro- or anti-tumorigenic factors. Among the various types of cytokines produced by tumor cells, interleukin-8 (IL-8, also known as CXCL8) was thought to alter leukocyte infiltration in the TME, thus resulting in the accumulation of pro-tumorigenic immune cells^6^. With the progression of glioma, the blood-brain barrier is compromised, thus resulting in the infiltration of circulating immune cells in the TME under certain chemoattractants^7^.

Neutrophils, the first cells that accumulate in inflammatory and damaged tissues^8^, are involved in different stages of oncogenic biological processes including tumor initiation, proliferation, or metastatic spreading^9^. The presence of tumor-infiltrating neutrophils (TINs) correlates with poor patient outcomes^10,11^. Glioblastomas with increased TINs activity exhibit aggressive tumor progression^12^. TINs promote glioma progression by releasing factors that remodel the TME, however, the underlying mechanisms remain to be uncovered.

Neutrophils exert their functions mainly through chemotaxis, phagocytosis, degranulation, and formation of neutrophil extracellular traps (NETs)^13^. NETs are web-like structures containing decondensed DNA filaments and granular contents extruded by neutrophils under certain stimuli^14^, and they induce a novel type of cell death that is different from apoptosis and necrosis^15^. NETs also contain a rich source of pro-inflammatory molecules and have been implicated in various sterile inflammatory conditions. Although NETs play important roles in the host defense against pathogens, excessive NETs formation is linked to various neutrophil-related pathologies, including thrombosis, autoimmunity, and metabolic disease, in a ROS-dependent manner^16–19^. Recent evidences indicate that NETs can awaken dormant cancer cells and promote cancer growth and metastasis^20,21^. Clinical studies suggest that elevated NETs are predictive of poor survival among patients with cancer^22^. To date, whether NETs are present in the glioma microenvironment and have a role in promoting glioma progression remain undetermined.

## Materials and methods

### Clinical datasets and tissue specimens

This study included 122 patients with glioma (WHO grade II, *n* = 37; grade III, *n* = 30; grade IV, *n* = 55) who received surgery between January 2012 and December 2019 at the Department of Neurosurgery at the Second Affiliated Hospital of Harbin Medical University. Clinicopathological data were collected as described in our previous study^23–25^. Surgically removed samples were confirmed by 2 pathologists. Full pre-treatment blood counts were performed on all patients as well as healthy volunteers (as the control group, *n* = 30). Informed consent was obtained from patients involved in this study. The study protocol was approved by the Clinical Research Ethics Committee of the Second Affiliated Hospital of Harbin Medical University (Approval No. KY2017-068), and all investigations were conducted in accordance with the Declaration of Helsinki.

### Cell culture

The human glioma cell line LN229 was purchased from the Chinese Academy of Sciences Cell Bank. HG7 primary cells were obtained from an adult female patient who previously had GBM^24^. All tumor cells were cultured in Dulbecco’s modified Eagle’s medium/F12 (Corning, Armonk, NY, USA) supplemented with 10% fetal bovine serum (BD Biosciences, San Jose, CA, USA) and 1% antibiotics (Sigma, St. Louis, MO, USA) at 37 °C in a humidified atmosphere with 5% CO_2_ and 95% air.

Neutrophils were isolated as described in our previous study^19^. Isolated neutrophils were cultured in RPMI 1640 medium (Corning, Armonk, NY, USA) with or without 10% fetal bovine serum. The neutrophil viability was > 95%, as assessed with trypan blue dye exclusion assays, and the neutrophil purity was > 98%, as assessed with Giemsa staining.

### Immunohistochemistry (IHC) assay

Briefly, 3-µm paraffin-embedded tissue sections were deparaffinized, rehydrated, treated with 0.3% hydrogen peroxide, and processed for antigen retrieval with heat induction for approximately 10 min^26^. Primary antibodies to the following proteins were used for IHC staining: CD66b (1:100, Proteintech Group, Chicago, IL, USA), neutrophil elastase (NE) (1:100, Abcam, Cambridge, Cambridgeshire, United Kingdom), and IL-8 (1:100, Affinity Biosciences, Cincinnati, OH, USA). Tissues were then incubated with peroxidase conjugated goat anti-mouse/rabbit IgG secondary antibody (ZSGB-bio, Beijing, China). The slides were finally stained with 3,3´-diaminoben-zidine (DAB substrate kit, ZSGB-bio, Beijing, China) and counterstained with hematoxylin. In negative controls, the primary antibody was replaced with phosphate buffered saline. The specimens were analyzed under a light microscope (Nikon, Tokyo, Japan) by pathologists.

Semiquantitative analysis was performed according to IHC scores^24,27^. The proportion of stained cell counts per field was scored as 0, 1, 2, and 3 if there was no positive staining, < 10% positive staining, 10%–30% positive staining, and > 30% positive staining, respectively. IHC scoring of cytokine expression was classified into 4 categories (0, 1, 2, and 3) according to the staining intensity (none, weak, moderate, and strong).

### Immunofluorescence assays

Tumor cells (LN229 or HG7, 1 × 10^5^) with or without treatments were seeded on coverslips and allowed to grow overnight. Isolated neutrophils (5 × 10^5^) with or without treatments were seeded on coverslips precoated with 0.001% poly-L-lysine for 3 h. Cells were then fixed with 4% paraformaldehyde for 30 min at room temperature, permeabilized with 0.5% Triton X-100 for 5 min, blocked with 50% goat serum in phosphate buffered saline for 30 min at 37 °C, and incubated with primary antibody against NE (1:100, Abcam, Cambridge, Cambridgeshire, United Kingdom), myeloperoxidase (MPO; 1:100, Proteintech Group, Chicago, IL, USA), citH3 (1:100, Abcam, Cambridge, Cambridgeshire, United Kingdom), CXCR2 (1:100, Abcam, Cambridge, Cambridgeshire, United Kingdom), Ki67 (1:100, Proteintech Group, Chicago, IL, USA), p65 (1:100, Proteintech Group, Chicago, IL, USA), RAGE (1:100, Abcam, Cambridge, Cambridgeshire, United Kingdom), or IL-8 (1:100, Affinity Biosciences, Cincinnati, OH, USA) in blocking buffer overnight at 4 °C. Cells were washed and incubated with Alexa Fluor 594- or 488-conjugated secondary antibody (1:1000, Life Technologies, Waltham, MA, USA). TRITC-labeled Phalloidin (1:200, Yeasen, Shanghai, China) was used to stain the F-actin in the cells. For labeling of DNA, cells were counterstained with DAPI (5 µg/mL, Invitrogen, Carlsbad, CA, USA). The images were acquired with a fluorescence microscope (ECLIPSE Ti, Nikon, Tokyo, Japan).

For immunofluorescence staining of glioma tissues, the tissue sections were deparaffinized, rehydrated, and processed with antigen retrieval as described above. Slides were then incubated with primary antibodies against MPO (1:100, Proteintech, Chicago, IL, USA) and citH3 (1:100, Abcam, Cambridge, Cambridgeshire, United Kingdom) followed by Alexa Fluor 594- or 488-conjugated secondary antibodies (1:1000, Life Technologies, Waltham, MA, USA). Tissue sections were counterstained with DAPI (5 µg/mL, Invitrogen, Carlsbad, CA, USA) and visualized under a fluorescence microscope.

### Cell proliferation and colony formation assays

A Cell-Counting Kit 8 (CCK-8; Dojindo Corp., Kumamoto, Japan) was used to test relative cell growth for different time intervals (0, 24, 48, and 72 h) according to the manufacturer’s instructions. The absorbance (optical density at 450 nm) of each 96-well plate was measured with a Tecan infinite F50 microplate reader. For colony formation assays, glioma cells were seeded in 6-well plates (500 cells per well) with the indicated treatments for 2 weeks. Cell colonies were fixed with 4% formaldehyde and stained with 0.1% crystal violet for 10 min, and the number of colonies was counted. All assays were performed in triplicate.

### Cell invasion and wound healing assays

Cell invasion assays were performed with the Boyden chamber invasion method^21^. A Boyden chamber (Corning, NY, USA) containing 8 µm pores was coated with Matrigel (Corning, NY, USA). Glioma cells (5 × 10^4^) were seeded into the upper chamber, and PMA-primed NETs-formation neutrophils (2.5 × 10^5^) on coverslips were seeded in the lower chamber with or without the indicated treatments. After incubation in 37 °C under 5% CO_2_ and 95% air, the non-invading tumor cells were wiped off the upper surface of the membrane, and the cells on the bottom side of the membrane were fixed in methanol for 10 min, then stained with crystal violet. The invading cells were counted under a light microscope.

For wound healing assays, tumor cells were grown to 70%–80% confluence on a 6-well tissue culture plate. Cell monolayers were manually wounded by scraping cells with a P200 pipette tip. Debris was removed, and the edge of the scratch was smoothed by washing the cells once with growth medium. Cells were then cultured in culture medium containing a lower percentage of serum (1%) to minimize the effect of cell proliferation on migration, and were then treated as indicated. The method was consistent with that in previous study^28,29^. The wound width was measured at different time intervals (12, 24, or 48 h) to evaluate the wound healing ability of glioma cells.

### Reverse transcription-polymerase chain reaction (RT-PCR)

Total RNA was extracted with TRIzol^®^ reagent (Life Technologies, Waltham, MA, USA). The cDNAs were synthesized with a Transcriptor First Strand cDNA Synthesis Kit (Roche, Basel, Switzerland) according to the manufacturer’s instructions. Conventional PCR was performed with a Bio-Rad system, and 1% agarose gel electrophoresis was used to detect the PCR products. The primer sequences used were as follows:

IL-8 forward: 5′-CTGCGCCAACACAGAAATTA-3′

IL-8 reverse: 5′-CATCTGGCAACCCTACAACA-3′

β-actin forward: 5′-CTCGCCTTTGCCGATCC-3′

β-actin reverse: 5′-GAATCCTTCTGACCCATGCC-3′.

β-actin was used as the reference control.

### Western blot

Western blot assays were performed with standard procedures^30^. Briefly, cells with or without treatments were harvested and lysed with RIPA buffer (Beyotime, Shanghai, China) with the proteinase inhibitor PMSF (1 mM) and a phosphatase inhibitor cocktail (1 mM, Roche, Basel, Switzerland). The protein concentration was detected with a BCA Protein Assay Kit (Beyotime, Shanghai, China). Equal amounts of proteins (20 µg/sample) were subjected to sodium dodecyl sulfate polyacrylamide gel electrophoresis and transferred to polyvinylidene difluoride membranes. After being blocked with 5% BSA in TBS-Tween, the membranes were incubated with primary antibodies overnight at 4 °C, followed by horseradish-peroxidase-conjugated secondary antibodies (1:4000, ZSGB-BIO, Beijing, China). Primary antibodies including anti-Cyclin D1 (Proteintech Group, Chicago, IL, USA), anti-Cyclin D2 (Proteintech Group, Chicago, IL, USA), anti-phospho-ERK1/2 (Cell Signaling Technology, CST, Massachusetts, USA), ERK1/2 (CST, Massachusetts, USA), anti-phospho-PI3K (CST, Massachusetts, USA), anti-PI3K (CST, Massachusetts, USA), anti-phospho-AKT (CST, Massachusetts, USA), anti-AKT (CST, Massachusetts, USA), and anti-β-actin (ZSGB-BIO, Beijing, China) were used at a dilution of 1:1000. Specific bands were detected with ECL Western blot substrate (Beyotime, Shanghai, China) with a ChemiDoc^TM^ MP Imaging System (Bio-Rad, Berkeley, CA, USA).

### Flow cytometry

Flow cytometry assays were conducted to detect relative changes in ROS in neutrophils^19,31^. Briefly, isolated neutrophils were resuspended in Hank’s solution (Solarbio, Beijing, China) at a concentration of 1 × 10^6^ cells/mL, and loaded with the fluorescent probe 2, 7-dichlorodihydrofluorescein diacetate (DCFH2-DA, 10 µM, Beyotime, Shanghai, China). After 3 washes to remove unloaded probe, cells were incubated with the indicated treatments. DCF relative fluorescence intensities in cells were quantified with flow cytometry (Becton Dickinson FACSCanto™ II, USA). Data were obtained in BD FACSDiva Software and analyzed in FlowJo X Software.

### Chemotaxis assays

Chemotaxis of isolated neutrophils was assayed in triplicate with a modified Boyden chamber with 8.0 µm pores (Corning, NY, USA). Freshly isolated neutrophils (1 × 10^6^ cells/mL) were resuspended in chemotaxis buffer (RPMI 1640, Corning, Armonk, NY, USA, with 10 mM HEPES and 0.5% BSA). Then 100 µL of neutrophils was placed in the upper chamber, and the lower chamber was filled with 500 µL of chemokine buffer [NETs-primed tumor cell conditioned medium (CM) or different concentrations of IL-8]. Cells that migrated into the lower chamber were manually quantified with a Neubauer improved cell counting chamber. The chemotactic index was evaluated as the ratio of migrated cells in each experimental condition to those in control buffer.

### Enzyme-linked immunosorbent assay (ELISA)

Cytokine levels in conditioned medium were determined with a quantitative sandwich enzyme immunoassay technique (IL-8 ELISA kit, Jianglai, Shanghai, China) according to the manufacturer’s instructions. Briefly, CM without phenol red was centrifuged for 20 min at 1,000 g. Then 50 µL of standard solution or 10× diluted samples were pipetted into the wells of 96-well plates precoated with anti-IL-8 monoclonal antibody, and a horseradish-peroxidase-labeled detection antibody was added to the wells. 3,3′,5,5′-tetramethylbenzidine (TMB) substrate was used to develop the color, and reactions were stopped with stop solution. The optical density was acquired with a microplate reader at a wavelength of 450 nm. Results were calculated according to the standard curve.

### Statistical analysis

Statistical results are presented as the mean ± SD. Student’s *t*-test was used to assess differences in the variable groups. Overall survival curves were used to describe the survival distributions, and the log-rank test was used for comparison. Chi-squared test was used to evaluate statistical differences in IHC staining of cytokines between groups. R packages, such as ggplot2 and KMsurv were used to produce figures. All statistical analyses were performed in SPSS 19.0 software (SPSS, Chicago, IL, USA) or GraphPad Prime 8.0 (La Jolla, CA, USA). *P* values less than 0.05 were considered statistically significant.

## Results

### Neutrophil infiltration and NETs formation are induced in high-grade glioma

**Figure 1:**
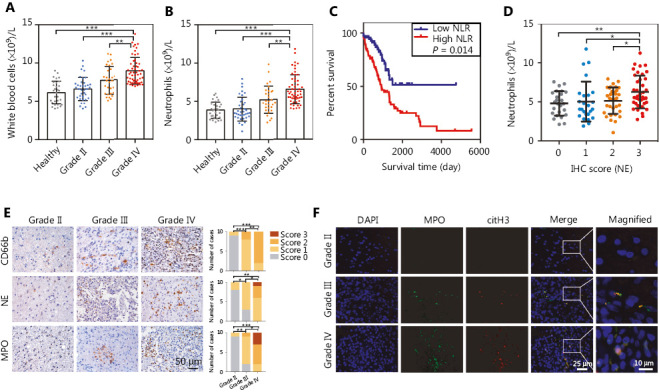
The infiltration of neutrophils and the formation of neutrophil extracellular traps (NETs) are associated with the malignancy of gliomas. (A) White blood cell counts of samples from healthy patients and patients with different WHO grades of gliomas. (B) Neutrophil cell counts of samples from healthy patients and patients with gliomas in different grades. (C) Kaplan–Meier survival analysis of patients with low neutrophil to lymphocyte ratio (NLR) and those with high NLR. (D) Neutrophil cell counts of samples with different immunohistochemistry (IHC) scores of NE. (E) IHC assays of CD66b, NE, and MPO in patients with different grades of gliomas (left panel), and corresponding IHC scores (right panel). (F) Immunofluorescence assays of MPO and citH3 in patients with different WHO grades of glioma. Significant results are presented as * *P* < 0.05, ** *P* < 0.01, or *** *P* < 0.001.

Activation and recruitment of neutrophils to tumor sites have been considered to correlate with greater tumor malignancy in several types of cancer^32,33^. In the present study, we examined the relationship between circulating neutrophil counts and glioma malignancy. A total of 122 patients with glioma (WHO grade II, *n* = 37; grade III, *n* = 30; grade IV, *n* = 55) and 30 healthy volunteers were enrolled in our study. We found that the number of circulating pretreatment leukocytes was significantly higher in patients with glioma than in people in the healthy control group, and the highest level was found in grade IV (**Figure 1A**, Healthy *vs.* Grade IV: *P* = 1.28E-10, Grade II *vs.* Grade IV: *P* = 3.81E-09, Grade III *vs.* Grade IV: *P* = 4.97E-03). Given that the elevation in resting white blood cells was unchanged, the elevated number of circulating neutrophils primarily contributed to this phenomenon (**Figure 1B**, Healthy *vs.* Grade IV: *P* = 2.90E-10, Grade II *vs.* Grade IV: *P* = 1.34E-09, Grade III *vs.* Grade IV: *P* = 2.51E-03). To further evaluate the correlation between pretreatment neutrophil counts and the prognosis of glioma patients, we estimated the survival of glioma patients with different neutrophil to lymphocyte ratio (NLR). Patients with high NLR (≥ 4) exhibited a poorer survival rate than those with low NLR (< 4) (**Figure 1C**, *P* = 1.40E-02). We also found a positive correlation between the circulating neutrophil count and the infiltrated neutrophil scores (**Figure 1D**, IHC score 0 *vs.* IHC score 3: *P* = 4.28E-03, IHC score 1 *vs.* IHC score 3: *P* = 3.92E-02, IHC score 2 *vs.* IHC score 3: *P* = 1.68E-02).

To clarify the involvement of neutrophils in promoting glioma progression, we analyzed the infiltration of neutrophils in glioma tissue by using IHC. CD66b, NE and MPO, defined as neutrophil markers, had elevated expression in grade IV glioma tissues compared with grade II or grade III glioma tissues (**Figure 1E**, CD66b: Grade II *vs.* Grade III: *P* = 3.00E-04, Grade II *vs.* Grade IV: *P* = 2.00E-04, Grade III *vs.* Grade IV: *P* = 7.30E-03; NE: Grade II *vs.* Grade III: *P* = 2.46E-02, Grade II *vs.* Grade IV: *P* = 2.90E-03, Grade III *vs.* Grade IV: *P* = 4.88E-02; MPO: Grade II *vs.* Grade III: *P* = 6.90E-03, Grade II *vs.* Grade IV: *P* = 6.00E-04, Grade III *vs.* Grade IV: *P* = 1.51E-02). Immunofluorescence analysis revealed higher levels of NETs in grade IV glioma tissues than in grade II or grade III glioma tissues, as determined by staining for MPO and citrullinated histone H3 (citH3) (**Figure 1F**). These data indicated that neutrophil infiltration and NETs formation were significantly associated with increased glioma malignancy.

### NETs secreted by TINs are involved in glioblastoma cell proliferation and invasion

**Figure 2:**
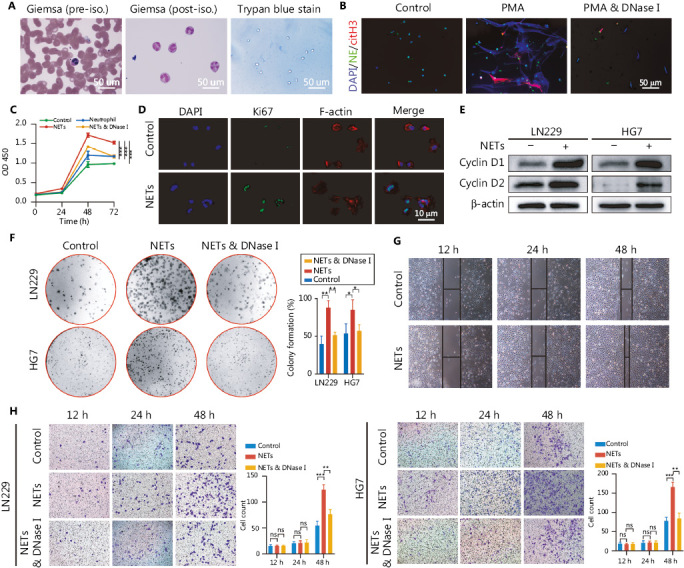
Neutrophil extracellular traps (NETs) promote proliferation, migration, and invasion of glioblastoma cells. (A) Isolation of neutrophils from blood samples of healthy volunteers. (B) Immunofluorescence assay of the confirmation of NETs formation. (C) CCK-8 assays of glioma cells treated with neutrophils, NETs, or NETs combined with DNase I (NETs & DNase I). (D) Immunofluorescence assays of Ki67 in glioma cells treated with or without NETs. (E) Western blot validation of cyclin D1 and cyclin D2 in LN229 and HG7 cells treated with or without NETs. (F) Colony formation assays of LN229 and HG7 cells treated with NETs or NETs combined with DNase I (NETs & DNase I). The statistics are shown in the histogram. (G) Wound-healing assays of LN229 cells treated with or without NETs. (H) Transwell invasion assays of LN229 and HG7 cells treated with NETs or NETs combined with DNase I (NETs & DNase I). The statistics are shown in the histogram. Significant results are presented as * *P* < 0.05, ** *P* < 0.01, or *** *P* < 0.001. Non-significant results are presented as ns.

NETs are mesh-like structures extruded by activated neutrophils in certain conditions. Fresh neutrophils isolated from healthy volunteers were primed with phorbol myristate acetate (PMA, 30 nM) to produce NETs (**Figure 2A**), and the formation of NETs was confirmed by immunofluorescence staining (**Figure 2B**). To explore the potential implications of NETs in glioma progression, we then co-cultured neutrophils, NETs, or NETs and DNase I (100 U/mL, Sigma, Saint Louis, MO, USA) with glioma cells (LN229). In CCK-8 assays, NETs significantly promoted the glioma cell proliferation rate, and this effect was abrogated in the NETs and DNase I group, because the NETs were digested by DNase I (**Figure 2C**, Control *vs.* NETs: *P* = 4.85E-05, Neutrophil *vs.* NETs: *P* = 1.94E-04, NETs combined with DNase I *vs.* NETs: *P* = 3.49E-04). In agreement with this effect, NETs incubation elevated the expression of the proliferation markers Ki67, Cyclin D1, and Cyclin D2 in glioma cells (**Figure 2D** and **2E**). Colony formation assays also indicated an oncogenic effect of NETs on glioma cells (LN229 and HG7) (**Figure 2F**, LN229: Control *vs.* NETs: *P* = 3.87E-03, NETs combined with DNase I *vs.* NETs: *P* = 3.16E-03; HG7: Control *vs.* NETs *P* = 4.30E-02, NETs combined with DNase I *vs.* NETs: *P* = 3.79E-02). Wound healing assays demonstrated that NETs enhanced the migration ability of glioma cells (**Figure 2G**). To investigate the effect of NETs on glioma cell invasiveness, we performed Transwell assays by seeding PMA-primed neutrophils in the lower chamber, to form NETs, and glioma cells in the upper chamber. As shown in **Figure 2H**, NETs significantly promoted tumor cell invasiveness, and this phenomenon was suppressed by NETs digestion with DNase I (LN229: 12 h, Control *vs.* NETs: *P* = 8.54E-01, NETs combined with DNase I *vs.* NETs: *P* = 7.68E-01, 24 h, Control *vs.* NETs: *P* = 8.16E-01, NETs combined with DNase I *vs.* NETs: *P* = 8.59E-01, 48 h, Control *vs.* NETs: *P* = 6.58E-04, NETs combined with DNase I *vs.* NETs: *P* = 3.21E-03; HG7: 12 h, Control *vs.* NETs: *P* = 6.53E-01, NETs combined with DNase I *vs.* NETs: *P* = 8.15E-01, 24 h, Control *vs.* NETs: *P* = 8.33E-01, NETs combined with DNase I *vs.* NETs: *P* = 9.07E-01, 48 h, Control *vs.* NETs: *P* = 5.59E-04, NETs combined with DNase I *vs.* NETs: *P* = 1.44E-03).

### HMGB1 derived from NETs binds to RAGE and activates the NF-κB signaling pathway in glioblastoma

**Figure 3:**
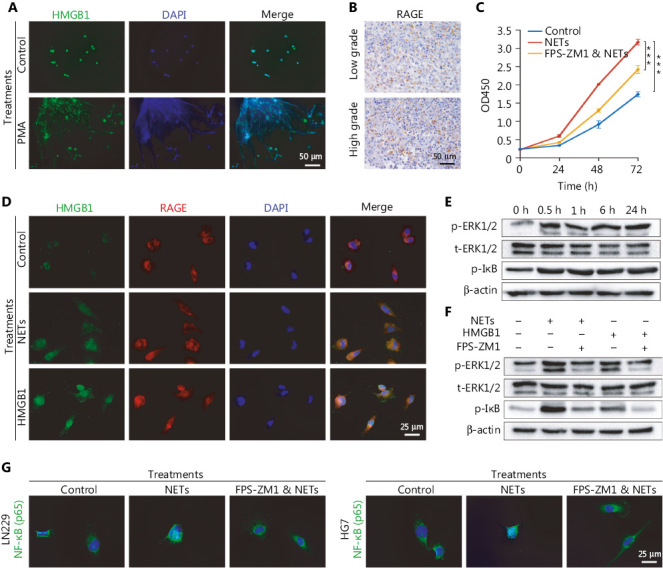
Neutrophil extracellular traps (NETs) mediate the malignancy of glioma cells *via* the HMGB1/RAGE/NF-κB axis. (A) Identification of HMGB1 by immunofluorescence assays on NETs. (B) The different RAGE expression in low-grade gliomas and high-grade gliomas. (C) CCK-8 assay of gliomas treated with NETs or NETs combined with FPS-ZM1 (FPS-ZM1 & NETs). (D) Immunofluorescence assays of HMGB1 and RAGE in glioma cells treated with NETs or recombinant HMGB1. (E) Expression of p-ERK1/2, t-ERK1/2 and p-IκB in glioma cells treated with recombinant HMGB1 for different time periods. (F) Expression of p-ERK1/2, t-ERK1/2, and p-IκB in glioma cells treated with NETs, recombinant HMGB1 or FPS-ZM1. (G) Nuclear translocation of NF-κB in LN229 and HG7 cells treated with NETs or NETs combined with FPS-ZM1 (FPS-ZM1 & NETs). Significant results are presented as *** *P* < 0.001.

NETs formation can result in the extracellular exposure of granular and nuclear proteins, of which HMGB1 is considered a key mediator in chronic inflammation process^34,35^. HMGB1 has also been reported to be involved in stem cell proliferation and migration^36,37^. Immunofluorescence staining results demonstrated that the mesh-like structures of NETs were decorated with HMGB1 (**Figure 3A**). Because RAGE is the major receptor for HMGB1 in mediating chronic sterile inflammation^38^, we proposed that NETs might exert oncogenic effects in an HMGB1/RAGE dependent manner in glioma. As shown in **Figure 3B**, RAGE expression was detected in glioma tissues by IHC, and intense RAGE staining was found in high-grade glioma. We then co-cultured NETs and LN229 cells with or without RAGE inhibitor (FPS-ZM1, 10 µM, Merck, Darmstadt, Germany) to block RAGE’s binding ability, and assessed the cell proliferation rate with CCK-8 assays. We found that FPS-ZM1 clearly suppressed NETs-induced tumor cell proliferation (**Figure 3C**, Control *vs.* NETs: *P* = 1.84E-05, FPS-ZM1 combined with NETs *vs.* NETs: *P* = 6.58E-04).

To further demonstrate the binding of HMGB1 to RAGE on glioma cells, we incubated LN229 cells with NETs or recombinant HMGB1 (1 µg/mL, Sigma, St. Louis, MO, USA). The binding ability was confirmed by immunofluorescence staining (**Figure 3D**). We found that recombinant HMGB1 treatment increased the phosphorylation levels of extracellular regulated protein kinases (ERK1/2) and inhibitor of kappa-B (IκB) from 0.5 h after treatments in LN229 cells (**Figure 3E**). Moreover, phosphorylation of ERK1/2 and IκB mediated by NETs or HMGB1 was abrogated by blocking RAGE on glioma cells (**Figure 3F**). These effects were further examined by detecting NF-κB nuclear translocation through immunofluorescence staining (**Figure 3G**).

### NETs stimulate the NF-κB signaling pathway to promote IL-8 secretion in glioblastoma

**Figure 4:**
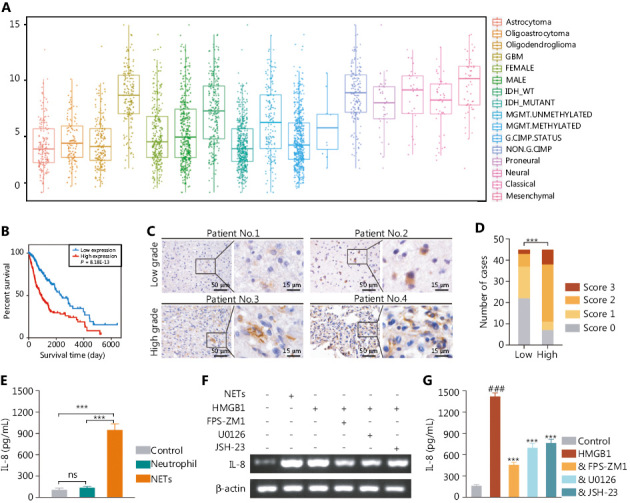
The IL-8 secretion of glioma cells is promoted *via* the HMGB1/RAGE/NF-κB axis. (A) IL-8 expression in different types of gliomas in the TCGA RNA-seq dataset. (B) Kaplan–Meier survival curve of TCGA glioma patients with low or high expression of IL-8. (C) Immunohistochemistry (IHC) assays of IL-8 in patients with low-grade or high-grade gliomas. (D) IHC score of IL-8 in low-grade or high-grade gliomas. (E) IL-8 secretion detected with ELISA in glioma cells treated with neutrophils or NETs. (F) The RNA level of IL-8 in LN229 cells treated with NETs, recombinant HMGB1, FPS-ZM1, U0126, or JSH-23. (G) IL-8 secretion detected with ELISA in glioma cells treated with or without FPS-ZM1, U0126, or JSH-23, followed by recombinant HMGB1 treatment (&FPS-ZM1, &U0126, or &JSH-23). Significant results are presented as *** *P* < 0.001, or ### *P* < 0.001. Non-significant results are presented as ns.

Aberrant activation of chronic inflammatory responses in the TME is a hallmark of tumor progression^39^. NF-κB acts as a key transcription factor for cytokines that mediate tumor glioma progression, including IL-8, which is a proinflammatory CXC chemokine mainly responsible for the recruitment of neutrophils^40,41^. Analysis of the IL-8 expression levels in The Cancer Genome Atlas (TCGA) glioma RNA-seq dataset (http://cancergenome.nih.gov/) showed that GBM and IDH wild-type patients had higher IL-8 expression levels than those in other groups (**Figure 4A**). Accordingly, the Kaplan–Meier survival curve indicated that patients with high-grade glioma have shorter survival times (**Figure 4B**, Low expression *vs.* High expression: *P* = 8.18E-13). The IL-8 expression in glioma tissues was also detected. As shown in **Figure 4C** and **4D**, patients with high-grade glioma exhibited significantly elevated levels of IL-8 staining (Low *vs.* High: *P* = 1.00E-06). To confirm that the elevated IL-8 was induced by NETs-primed glioma cells, we co-cultured LN229 cells with neutrophils or NETs, and detected IL-8 expression in CM by ELISA. The data clearly showed that LN229 cells co-cultured with NETs elevated IL-8 concentration, as compared with those co-cultured with neutrophils or control group (normal LN229 cell culture medium) (**Figure 4E**, Control *vs.* NETs: *P* = 8.36E-05, Neutrophil *vs.* NETs: *P* = 8.86E-05, Control *vs.* Neutrophil: *P* = 1.79E-01). To further investigate the involvement of the RAGE/ERK/NF-κB axis in IL-8 production, we used FPS-ZM1, U0126 (10 µM, ERK1/2 inhibitor, Sigma, St. Louis, MO, USA) and JSH-23 (25 µM, NF-κB inhibitor, Hycultec GmbH, Beutelsbach, Germany) to inhibit RAGE, ERK, and NF-κB activation, respectively. We found that the inhibition of the RAGE/ERK/NF-κB axis significantly decreased the NETs or HMGB1 induced IL-8 production in glioma cells at both mRNA and protein levels (**Figure 4F** and G, Control *vs.* HMGB1: *P* = 2.26E-06, HMGB1 combined with FPS-ZM1 *vs.* HMGB1: *P* = 1.15E-05, HMGB1 combined with U0126 *vs.* HMGB1: *P* = 9.89E-05, HMGB1 combined with JSH-23 *vs.* HMGB1: *P* = 1.04E-04). These results indicated that HMGB1 in NETs induced IL-8 secretion through the RAGE/ERK/NF-κB axis in glioma cells.

### IL-8 recruits neutrophils and mediates NETs formation ***via*** the PI3K/AKT/ROS axis in TINs

**Figure 5:**
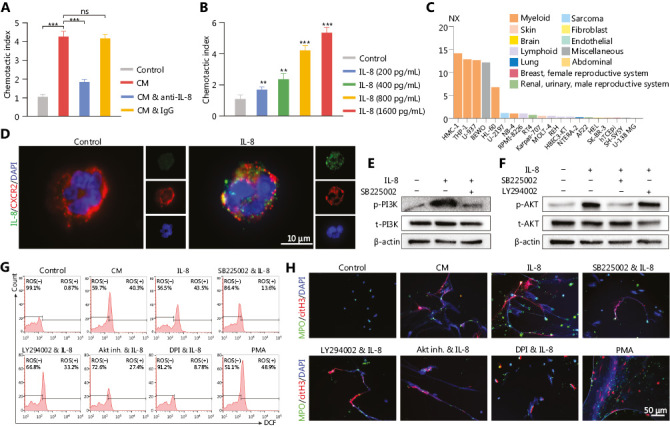
IL-8 recruits neutrophils and mediates neutrophil extracellular traps (NETs) formation. (A) Recruitment of neutrophils detected with chemotaxis assays with NETs-primed conditioned medium (CM), CM combined with anti-IL-8 antibody (CM & anti-IL-8), or CM combined with isotype IgG (CM & IgG). (B) Recruitment of neutrophils, detected with chemotaxis assays with different concentrations of IL-8. (C) Expression levels of CXCR2 in different cell lines. (D) Binding affinity of IL-8 and CXCR2 on neutrophils, detected with immunofluorescence assays. (E) Expression of p-PI3K and t-PI3K in neutrophils treated with IL-8 or IL-8 combined with SB225002 (200 nM, CXCR2 inhibitor, Calbiochem). (F) Expression of p-AKT and t-AKT in neutrophils treated with IL-8, SB225002, or LY294002 (5 µM, PI3K inhibitor, EMD, Billerica, MA). (G) ROS activity in neutrophils treated with CM, IL-8, IL-8 combined with SB225002 (SB225002 & IL-8), IL-8 combined with LY294002 (LY294002 & IL-8), IL-8 combined with AKT inhibitor 1/2 (Akt inh. & IL-8; Akt inh., 10 μM, Calbiochem, San Diego, CA), IL-8 combined with DPI (DPI & IL-8; 10 μM, NADPH oxidase inhibitor, Sigma, Saint Louis, MO, USA), or PMA (30 nM). (H) Detection of NETs formation by immunofluorescence in neutrophils treated with CM, IL-8, IL-8 combined with SB225002 (SB225002 & IL-8), IL-8 combined with LY294002 (LY294002 & IL-8), IL-8 combined with AKT inhibitor 1/2 (Akt inh. & IL-8), IL-8 combined with DPI (DPI & IL-8), or PMA. Significant results are presented as *** *P* < 0.01. Non-significant results are presented as ns.

Chemotaxis assays were performed to determine whether the infiltration of neutrophils in glioma tissue was due to the attraction of IL-8. NETs-primed LN229 cell CM exhibited a higher ability to attract neutrophils compared to the control group (cell culture medium only) (**Figure 5A**, control *vs.* CM: *P* = 7.17E-07, CM *vs.* CM combined with anti-IL-8: *P* = 3.86E-06, CM *vs.* CM combined with IgG: *P* = 5.92E-01). This effect was significantly repressed by the addition of anti-IL-8 neutralizing antibody into the CM, whereas the addition of isotype control antibody had no suppressive effect (**Figure 5A**). The neutrophil attraction effect of IL-8 was further confirmed that with higher concentrations of recombinant IL-8, the chemotaxis index was upregulated [**Figure 5B**, Control *vs.* IL-8 (200 pg/mL): *P* = 7.73E-03, Control *vs.* IL-8 (400 pg/mL): *P* = 1.09E-03, Control *vs.* IL-8 (800 pg/mL): *P* = 4.03E-06, Control *vs.* IL-8 (1600 pg mL): *P* = 1.06E-06]. CXCR2, a cell surface receptor of IL-8^42^, was primarily expressed on myeloid lineage cells, including neutrophils in the Protein Atlas dataset (https://www.proteinatlas.org) (**Figure 5C**). With freshly isolated neutrophils incubated with recombinant IL-8, we found that IL-8 colocalized with CXCR2 on neutrophils (**Figure 5D**).

**Figure 6:**
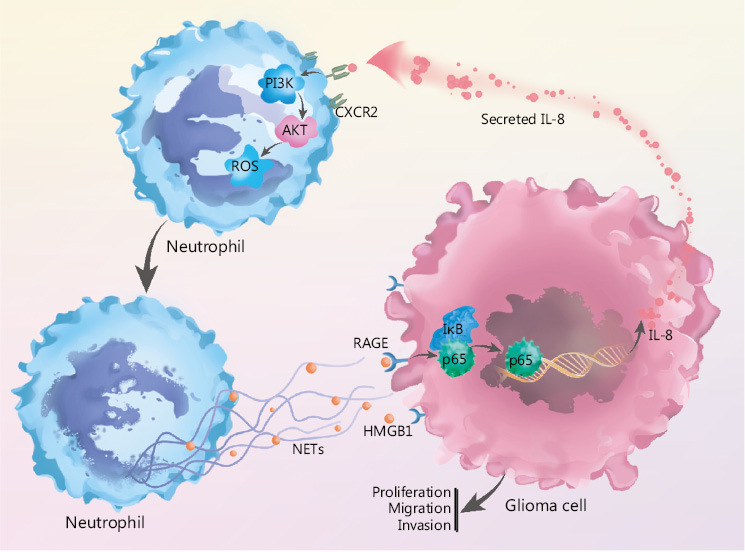
NETs play a role in the crosstalk between glioma progression and tumor microenvironment by regulating the HMGB1/RAGE/IL-8 axis is shown.

By incubating recombinant IL-8 with freshly isolated neutrophils, we observed elevated PI3K phosphorylation in neutrophils, and this effect was clearly abrogated by blocking CXCR2 (**Figure 5E**). In agreement with these results, we identified that IL-8 incubation led to the phosphorylation of AKT, a key mediator downstream of the PI3K signaling pathway (**Figure 5F**). We further explored the effect of NETs-primed glioma cell CM and recombinant IL-8 on ROS production in freshly isolated neutrophils. As shown in **Figure 5G**, the CM or IL-8 incubation resulted in robust production of ROS in neutrophils, and inhibition of the CXCR2/PI3K/AKT axis or NADPH oxidase reversed this effect, thus indicating their involvement in IL-8-induced ROS production. We also found that incubating NETs-primed glioma cell CM or recombinant IL-8 with freshly isolated neutrophils resulted in NETs formation, and this effect was suppressed by inhibition of CXCR2 or the PI3K/AKT/ROS axis (**Figure 5H**). These results advanced understanding of NETs in the crosstalk between glioma progression and the TME by regulating the HMGB1/RAGE/IL-8 axis (**Figure 6**).

## Discussion

Glioma is the most common brain tumor in adults and has high self-renewal ability, invasive capability, and drug resistance. Patients with GBM, the most malignant type of gliomas, have poor prognoses of no more than 15 months after comprehensive treatment with surgical removal^2^. In the past decade, the TME has received considerable attention, owing to the reciprocal interactions between TME cells and tumor cells^43^. Increasing evidence indicates that chronic inflammation promotes tumor initiation, progression, and metastasis by influencing the TME. The central nervous system has traditionally been recognized as an immune-privileged site, whereas recent data indicate that immune cells can traffic to the central nervous system when the blood-brain barrier is disrupted by tissue injury or inflammation caused by malignant gliomas^44^. The aberrant activation of inflammatory responses results in the production of ROS and induces tumor progression^4^.

Neutrophils are the most abundant type of white blood cells^45^, and TINs have been reported to be associated with decreased recurrence-free time and overall survival in renal carcinomas^10^, head and neck squamous cell carcinoma^46^, pancreatic adenocarcinomas^47^, and liver carcinoma^48^. In the present study, we found that TINs in the glioma microenvironment are significantly correlated with glioma grade. Patients with high NLR exhibit poorer survival than those with low NLR.

Through releasing factors that remodel the TME, TINs promote tumor progression^9^. NETs, produced by TINs, are complex extracellular structures composed of chromatin and specific proteins such as histones, MPO, cathepsin G, leukocyte proteinase 3 (PR3), and lysozyme C^49^. The enhanced expression of MPO and citH3 demonstrated increased NETs secretion in the glioblastoma microenvironment. Blocking NETs through multiple strategies significantly inhibits spontaneous metastasis of tumors^50^. With DNase I digestion, the increasing glioma cell proliferation and invasion induced by NETs were abrogated.

HMGB1 is an important component of NETs^51^ and is a highly conserved nuclear protein widely present in all cell types^52^. After being released into the extracellular environment and binding to certain receptors, HMGB1 mediates cell proliferation, survival, and migration^53^. In our present work, we found that the mesh-like structure of NETs was decorated with HMGB1, and the externalized HMGB1 interaction with RAGE on glioma cells led to the activation of the transcription factor NF-κB, which has been reported to be a key transcription factor affecting IL-8 expression^41^. We further demonstrated that IL-8 was highly expressed in patients with high-grade glioma, and that NETs induced IL-8 expression in glioma cells in a HMGB1- and RAGE/ERK/NF-κB axis-dependent manner.

IL-8, also known as CXCL8, is a proinflammatory CXC chemokine. Malignant tumor cells secrete IL-8 under certain environmental stresses, including hypoxia and chemotherapy agents^54^. IL-8 concentrations are significantly higher in many types of cancer than in healthy tissues, and have been found to correlate with tumor burden and prognosis^55,56^. Increasing evidence indicates that tumor-produced IL-8 can function in a paracrine manner to alter the composition of immune cells in the TME^6^. After IL-8 stimulation, endothelial cells begin an angiogenic process that delivers essential nutrients and oxygen to tumor cells^57^. Tumor-produced IL-8 tends to the increase of neutrophils or myeloid-derived suppressor cells (MDSCs) and leads to the induction of an immunosuppressive TME^55^. Consistently with these findings, patients with elevated levels of circulating MDSCs tend to have elevated serum IL-8 levels^58^. In this study, we found that high levels of IL-8 expression in glioma tissue were always accompanied by increased neutrophil infiltration, thus indicating an alteration in glioma TME. IL-8 signals through interaction with the G protein-coupled receptors CXCR1 or CXCR2^59^. Pharmacological inhibition of CXCR1 or CXCR2 represses neutrophil infiltration into tumor sites, thus resulting in repression of tumor growth^60^. Activation of G proteins determines the activation of the phosphoinositide 3-kinases (PI3K) and members of the RAS family^61^. The PI3K signaling pathway plays a pivotal role in mediating neutrophil chemotaxis and phagocytosis^62^. In our present work, we identified the expression of CXCR2 on isolated neutrophils and confirmed the binding of IL-8 to CXCR2. Additionally, we found that stimulating freshly isolated neutrophils with tumor-produced IL-8 resulted in the phosphorylation of PI3K and AKT, which further led to ROS generation and NETs formation.

## Conclusions

Together, our results provide evidences that TINs-formed NETs promote glioma cell proliferation, migration, and invasion. Those tumor cells in turn secrete IL-8, which increases neutrophil infiltration into tumor sites and primes neutrophils to form additional NETs. Eventually, this positive feedback loop results in a reciprocal interaction between NETs and tumor cells, alteration of the TME, and progression of glioma.
